# Health communication, information technology and the public’s attitude toward periodic general health examinations

**DOI:** 10.12688/f1000research.10508.1

**Published:** 2016-12-30

**Authors:** Quan-Hoang Vuong

**Affiliations:** 1 FPT University, Hanoi, Vietnam

**Keywords:** general health examination, subclinical screenings, information and communication technology, healthcare subsidy

## Abstract

*Background: *Periodic general health examinations (GHEs) are gradually becoming more popular as they employ subclinical screenings, as a means of early detection. This study considers the effect of information technology (IT), health communications and the public’s attitude towards GHEs in Vietnam. *Methods: *A total of 2,068 valid observations were obtained from a survey in Hanoi and its surrounding areas. *Results: *In total, 42.12% of participants stated that they were willing to use IT applications to recognise illness symptoms, and nearly 2/3 of them rated the healthcare quality at average level or below. *Discussion: *The data, which was processed by the BCL model, showed that IT applications (apps) reduce hesitation toward GHEs; however, older people seem to have less confidence in using these apps. Health communications and government’s subsidy also increased the likelihood of people attending periodic GHEs. The probability of early check-ups where there is a cash subsidy could reach approximately 80%.

## Introduction

Nowadays, people tend to avoid taking clinical treatments, instead, they prefer having subclinical tests and screenings as preventive medicine ^1– 4^. Using mobile applications (apps) in medical care is now becoming more popular thanks to the proliferation of information technology (IT) ^5– 8^ ( http://www.mobihealthnews.com/4740/physician-smartphone-adoption-rate-to-reach-81-in-2012). As of 2012, there were 114 countries all over the world using mobile technology in medical care ^9^, and a total of 165,000 mobile health apps were on the market in 2015 ( http://www.imedicalapps.com/2015/09/ims-health-apps-report/), which were used in various different specialities from orthopaedics to cardiology ^10, 11^. West (2012) indicated that mobile technology was helping with chronic disease management, empowering the elderly and expectant mothers, reminding people to take medication at the proper time, extending services to underserved areas, and improving health outcomes and medical system efficiency ^9^. In the same vein, some other studies also underscored the effectiveness of these apps in remote treatment in developing countries ^12– 14^. This efficiency was allegedly because they assisted faster decision making, transmitting messages more quickly and therefore saving money ^9, 15^. However, Buijink *et al* argued that almost all these mobile apps lacked authenticity or professional involvement, which could result in a wrong diagnosis, which may cause harm to the users ^10, 18^.

Due to the above limitations, many people still prefer to have direct clinical check-ups with doctors for prevention and early detection through periodic general health examinations (GHEs). However, this usually costs a substantial amount of money for clinical treatment, subclinical screenings or preventive services that we use ^19– 21^. People are more worried about increasing healthcare costs than being unemployed or terrorism ^22^, since the financial burden could push them into poverty or even destitution ^23^. Yet, the quality of medical services is still not compatible with what the patient’s pay for, as the majority of patients have low satisfaction with doctors and nursing care, especially with waiting time ^24, 25^. Responsiveness is usually the top factor that patients expect ^26, 27^, but the reality still falls far short of their expectations ^24, 25, 28, 29^. Those who have a high education background are more likely to demand higher standards on medical quality ^30, 31^. Conversely, the elderly tend to be more easily satisfied, with evidence from different countries in the world ^32, 33^.

Health communications, usually delivering case information, social consequences and policy messages, also have a certain influence on peoples’ behaviours and attitudes toward medical services ^33^. Vivid, fearful and credible messages are apparently more persuasive ^22, 33– 35^. Younger people prefer social consequence communications, whereas older people are more influenced by physical consequences ^33^. Furthermore, women respond to emotional messages with social consequences for oneself or health consequences to near and dear ones, whereas men are more influenced by unemotional messages that emphasise personal physical health consequences ^33^.

The majority of Vietnamese households still take advice from relatives or friends rather than from professionals on making clinical treatment-related decisions ^36^. Families are the primary units for health education across most countries, whatever the level of economic development, and help establish culturally engrained beliefs about health and illness ^37^. Family members and friends are huge sources of health information that can affect prevention, control and care activities ^38^. Moreover, the social networks surrounding each health consumer also have powerful influences on their health beliefs and behaviours ^39^. The quality of information and professional credibility are critical factors that help patients choose a healthcare provider ^40^. However, it is not productive to encourage people to seek early detection, diagnosis and treatment when they have limited access to care, which is a reality in many developing countries ^41^.

In this study, four models are employed to find out the influences of factors, including health communications, IT apps, age, education backgrounds, willingness/hesitations toward periodic GHE and government subsidies, on peoples’ attitude and behaviours toward preventive, subclinical or GHE decisions.

## Methods

### Survey characteristics

A survey was conducted by the research team from the office of Vuong & Associates ( http://www.vuongassociates.com/home), who directly interviewed people in the areas of Hanoi and Hung Yen (Vietnam) in the period between September and October 2016. The study was performed under a license granted by the joint Ethics Board of Hospital 125 Thai Thinh, Hanoi, and Vuong & Associates Research Board (V&A/07/2016; 15 September 2016). Written informed consent was obtained from the participants prior to starting the survey. The questions selected were fairly simple and easy to understand, which when coupled with the enthusiasm of the participants, led to straightforward interviews. The subjects of the survey were chosen completely randomly and there was no exclusion criteria. The obtained dataset contained 2,068 observations ( Dataset 1 ^42^).

Regarding the data collecting process, since the data sample is random, no specific criteria for selecting some groups of people, like gender or age or job, were imposed. The survey team targeted places where most people are willing to spend time to take part in the survey. The interviewing places were public and private hospitals, junior high and high schools and business offices around Hanoi. Each respondent was given 10 to 20 minutes for each questionnaire, and the survey took place after the participant had understood the research ethics, content of the survey and ways of responding to the questions. The full questionnaire was delivered in Vietnamese, with a clear statement of research ethics standards, and is provided in Supplementary File 1 (an English translation can be found in Supplementary File 2).

Apart from the basic descriptive statistics, the present study employed statistical methods of categorical data analysis for modelling baseline category logits (i.e., BCL models), with the existence of continuous variables, as provided in Table 2. The practical estimations of categorical data following BCL models follow ^23^.

### Data modelling

The data were entered into Microsoft Office Excel 2007, then processed by R (3.3.1). The estimates in the study were made using BCL logistic regression models ^23^ to predict the likelihood of a category of response variable *Y* in various conditions of predictor variable *x*.

The general equation of the baseline-categorical logit model is:

                                                              ln(π _j_( **x**)/π _J_( **x**)) = **α**_j_+ **β**_j_^’^ **x**,       j=1,…, J-1.

in which **x** is the independent variable; and *π*_j_( **x**)= *P*( *Y*= *j*/ **x**) is its probability. Thus *π*_j_= *P*( *Y _ij_*=1), with *Y* being the dependent variable.

In the logit model in consideration, the probability of an event is calculated as:

*π*_j_( **x**) = exp( **α**_*j*_+ **β**_*j*_^’^ **x**)/[1+ ^J-1^∑ _(h-1)_exp( **α**_j_+ **β**_j_^’^ **x**)]

with ∑ *_j_π _j_*( **x**) =1; α _*J*_ = 0 and β _*J*_ = 0; *n* is the number of observations in the sample, *j* is the categorical values of an observation *i* and *h* is a row in basic matrix *X _i_*, see 23. In the analysis, *z*-value and *p*-value are the bases to conclude the statistical significance of predictor variables in the models, with *P* < 0.05 being the conventional level of statistical significance required for a positive result.

## Results

### Sample characteristics

The sample totalled 2,068 participants, of which 1,510 had an educational level of university or above (73.02%). A total of 1,073 participants expressed hesitation toward attending GHEs because they do not think it is not urgent or important ( Table 1).

When seeing clinical signs, many respondents choose clinics as the first priority (43.04%), while 29.45% seek relatives or friends’ advice and 27.51% prefer to self-study. Furthermore, the majority (86.32%) are ready to pay for healthcare if the cost of a periodic GHE is less than VND 2 million.

Of the participants, 42.12% were willing to use mobile health apps if they are supposedly credible. If the apps reveal some health problems, 78.96% of participants may or will certainly go to the clinic to receive a check-up. Regarding the quality of medical services, most of the respondents expressed poor experiences; 1,291 participants scored the quality of medical services medium, while 60 scored it low.

Regarding peoples’ assessments of GHE quality, a scale of 5 (1 is lowest, 5 is highest) was used. “Respon” is the element that was assessed lowest among five elements (Response, Tangibility, Reliability, Assurance and Empathy) with 3.38 points (Tangibility 3.61 points; Reliability 3.57 points; Assurance 3.69 points; and Empathy 3.47 points) and is 0.17 points lower than the composite point (3.55). On the contrary, when it comes to health communications, ‘sufficiency of information’ achieved 3.01 points (95% CI: 2.96 - 3.06), which is the highest among the four components constituting the factor of health communications, apart from ‘the efficiency of health communications’, which is 0.18 points higher than the average at 2.83 (the two other components are: the attractiveness (2.69 points) and emphasis of information (2.82 points)).

**Table 1.** Descriptive statistics concerning education background, motivation for attending GHEs, income and use of IT apps in survey participants.

Characteristics	N	%
**Education background (“Edu”)** Secondary or high school (“Hi”) University or higher (“Uni”)	558 1,510	26.98 73.02
**Hesitation due to non-urgency and unimportance (“NotImp”)** Yes No	1,073 995	51.89 48.11
**Readiness due to community subsidy (“ComSubsidy”)** Yes No	1,061 1,007	51.31 48.69
**Usage of subsidy (“UseMon”)** Spending all soon (“allsoon”) Spending part and saving the rest (“partly”) Taking the money and using it later (“later”)	1,286 311 471	62.19 15.04 22.77
**First choices as having illness symptoms (“StChoice”)** Clinic (“clinic”) Asking relatives or friends (“askrel”) Self-study (“selfstudy”)	890 609 569	43.04 29.45 27.51
**Affordable GHE costs** Less than VND 1 million (“low”) VND 1–2 million (“med”) Above VND 2 million (“hi”)	876 909 283	42.36 43.96 13.68
**Ready to use IT apps (“UseIT”)** Yes Maybe No	871 721 476	42.12 34.86 23.02
**Take GHE if IT apps show health problems (“AfterIT”)** Yes Maybe No	815 900 353	39.41 43.52 17.07
**Assessments toward GHE’s quality (“QualExam”)** From 1 to < 2 points (“low”) From 2 to < 4 points (“med”) From 4 to 5 points (“hi”)	60 1,291 717	2.90 62.43 34.67

*Note: Codes of variables used in R estimations in brackets

### Propensities toward periodic GHE

***Propensities toward the first choice when experiencing disease symptoms.*** Employing logistic regression estimations with the dependent variable “StChoice” against four independent variables “Edu”, “Age”, “Respon” and “PopularInfo”, introduced in Table 2, the results reported in Table 3 show that there are relationships between the choice people prioritise when they recognise their symptoms with age, educational background, physicians’ responsiveness and the sufficiency of health information.

**Table 2.** Descriptive statistics for continuous variables used in subsequent estimations.

Characteristics	Average	SD	CI
Age, years	29.17	10.09	28.74-29.60
Assessments of responsiveness (“Respon”)	3.38	1.260	3.33-3.43
Assessments of efficiency of health communications (“PopularInfo”)	2.80	1.180	2.75-2.85
Assessments of information sufficiency (“SuffInfo”)	3.01	1.170	2.96-3.06

*Note: Variables “Respon”, “PopularInfo” and “SuffInfo” have the lowest value of 1 and highest 5.

**Table 3.** Estimation results with response variable “StChoice” and predictors “Edu”, “Age”, “Respon” and “PopularInfo”.

	Intercept	“Edu”	“Age”	“Respon”	“PopularInfo”
		“Hi”			
	***β*_0_**	***β*_1_**	***β*_2_**	***β*_3_**	***β*_4_**
logit(askrel|selfstudy)	1.004*** [3.636]	0.712*** [4.844]	-0.025*** [-3.438]	-0.225*** [-4.709]	0.123* [2.398]
logit(clinic|selfstudy)	-0.673** [-2.656]	0.578*** [4.227]	0.026*** [4.372]	-0.067 [-1.502]	0.159*** [3.354]

Signif. codes: 0 ‘***’ 0.001 ‘**’ 0.01 ‘*’ 0.05 ‘.’ 0.1 ‘ ’ 1; z-value in square brackets; baseline category for: “Edu”=“Uni”. Residual deviance: 4304.03 on 4126 degrees of freedom.

( Eq.1) and ( Eq.2) are established based on Table 3 as follows:

        ln( *π*_askrel_/ *π*_selfstudy_) = 1.004 + 0.712× *Hi.Edu* – 0.025× *Age* – 0.225× *Respon* + 0.123× *PopularInfo*                (Eq.1)

        ln( *π*_clinic_/ *π*_selfstudy_) = –0.673 + 0.578× *Hi.Edu* + 0.026× *Age* – 0.067× *Respon* + 0.158× *PopularInfo*                (Eq.2)

From the two above formulas, the probability of a person aged 30, giving 3.38 points for doctors’ responsiveness and 2.08 points for the efficiency of health communications (average points), choosing to go to clinic as the first choice is:

*π*_clinic_= e ^-0.673+0.578+0.026×30-0.067×3.38+0.158×2.8^ **/**[1+ e ^-0.673+0.578+0.026×30-0.067×3.38+0.158×2.8^ + e ^(1.004+0.712-0.025×30-0.225×3.38+0.123×2.8)^] = 0.474

In the same manner, the probability calculated in the case that this person has a university or higher education background is 42.74%.

***Decision to attend periodic GHE after using IT apps.*** The results of logistic regression with the independent variables “Age”, “UseIT”, “PopularInfo” and the dependent variable “AfterIT” has shown the effect of age, the efficiency of health communications and the readiness to use IT health apps on the decision to attend GHE if the apps identify health problems.

From that, in ln( *π*_maybe_/ *π*_yes_), the intercept *β*_0_=1.624 ( *P*<0.001, *z*=6.833), the coefficient of “Age” *β*_1_=0.001 ( *P*<1, *z*=0.165); the coefficient of “UseIT” at “no” is *β _2_* =-1.744 ( *P*<0.001, *z*=-9.816) and at “yes” is *β _3_*=-2.558 ( *P*<0.001, *z*=-19.870). The coefficient of “PopularInfo” *β _4_*=-0.008 ( *P*<1, *z*=-0.169).

In ln( *π*_no_/ *π*_yes_), the intercept *β*_0_=-1.290 ( *P*<0.001, *z*=-3.785), the coefficient of “Age” *β*_1_=0.026 ( *P*<0.001, *z*=3.470); the coefficient of “UseIT” at “no” is *β*_2_=2.022 ( *P*<0.001, *z*=9.095) and at “yes” *β*_3_=-1.774 ( *P*<0.001, *z*=-6.859). For the coefficient “PopularInfo”, *β*_4_=-0.210 ( *P*<0.01, *z*=-3.094).

The two formulas below describe the relationships between the factors:

        ln( *π*_maybe_/ *π*_yes_) = 1.624 + 0.001× *Age* – 1.744× *no.UseIT* – 2.558× *yes.UseIT* – 0.008× *PopularInfo*                (Eq.3)

        ln(π _no_/π _yes_) = –1.290 + 0.026× *Age* + 2.022× *no.UseIT* – 1.774× *yes.UseIT* – 0.210× *PopularInfo*                (Eq.4)

Based on ( Eq.3) and ( Eq.4), we can calculate the probabilities of a patient taking GHE after IT apps reveal health problems with “Age”=30, “PopularInfo”=2.80 and “UseIT”=“yes” is 68.84%. In case “UseIT” = “no”, *π*_yes_=22.66%.

### Assessments of healthcare services’ quality associated with health communications

Employing a logistic regression model with the response “QualExam” and two continuous dependent variables “SuffInfo” and “PopularInfo”, the results are described as follows. In ln( *π*_hi_/ *π*_med_), the intercept *β*_0_=-1.525 ( *P*<0.001, *z*=-10.317), the coefficients of “SuffInfo” and “PopularInfo” are *β*_1_=0.114 ( *P*<0.05, *z*=2.298) and *β*_2_=0.204 ( *P*<0.001, *z*=4.169), respectively. In addition, for ln( *π*_low_/ *π*_med_), intercept *β*_0_=-1.454 ( *P*<0.001, *z*=-4.235), the coefficients of “SuffInfo” and “PopularInfo” are *β*_1_=-0.635( *P*<0.001, *z*=-4.080) and *β*_2_=-0.005 ( *P*<1, *z*=-0.035), respectively.

The two regression equations:

        ln(π _hi_/π _med_) = –1.525 + 0.114 × *SuffInfo* + 0.204 × *PopularInfo*                (Eq.5)

        ln(π _low_/π _med_) = –1.454 – 0.635 × *SuffInfo* – 0.005 × *PopularInfo*                (Eq.6)

### Propensities of attending GHEs with availability of healthcare subsidy

The correlation between the hesitation toward GHE, due to perceived non-urgency and unimportance, the readiness due to community subsidy, affordable costs and the usage of subsidy is confirmed with the results as follows: In ln( *π*_allsoon_/ *π*_partly_), the intercept β _0_=1.868 ( *P*<0.001, *z*=12.763), the coefficient of “NotImp” at “yes” is *β _1_*=-0.350 ( *P*<0.01, *z*=-2.706), the coefficient of “ComSubsidy” at “yes” is *β*_2_=0.097 ( *P*<1, *z*=0.751), the coefficient of “AffCost” at “hi” is *β*_3_=0.699 ( *P*<0.05, *z*=2.477) and at “low” is *β*_4_=-0.752 ( *P*<0.001, *z*=-5.490).

Likewise, in ln( *π*_later_/ *π*_partly_), the intercept *β*_0_=0.910 ( *P*<0.001, *z*=5.464), the coefficient of “NotImp” at “yes” is *β*_1_=0.303 ( *P*<0.05, *z*=1.989), the coefficient of “ComSubsidy” at “yes” is *β*_2_=-0.672 ( *P*<0.001, *z*=-4.459), and “AffCost” at “hi” is *β*_3_=0.790 ( *P*<0.01, *z*=2.622) and at “low” is *β*_4_=-0.916 ( *P*<0.001, *z*=-5.714).

Regression equations ( Eq.7) and ( Eq.8) are built based on the above results:

        ln( *π*_allsoon_/ *π*_partly_) = 0.910 + 0.303× *yes.NotImp* – 0.672× *yes.ComSubsidy* + 0.790 × *hi.AffCost* – 0.916× *low.AffCost*                (Eq.7)

        ln( *π*_later_/ *π*_partly_) = 1.868 – 0.350× *yes.NotImp* + 0.097× *yes.ComSubsidy* + 0.699 × *hi.AffCost* – 0.752× *low.AffCost*                (Eq.8)

From ( Eq.7) and ( Eq.8), the probability of a person using all of a subsidy soon being ready to participate in GHE, having no hesitation and willing to pay at high cost is calculated as follows:

*π*_allsoon _= e ^1.868+0.097+0.699^ **/**[1+ e ^1.868+0.097+0.699^ + e ^0.910-0.672+0.790^]=0.791

The same procedure could be used to compute other likelihoods ( Supplementary File 3).

Raw data gathered from the survey The data table used for providing descriptive statistics and preparing data subsets for statistical analysis (see also Supplementary Table 1). Click here for additional data file. Copyright: © 2016 Vuong QH 2016 Data associated with the article are available under the terms of the Creative Commons Zero "No rights reserved" data waiver (CC0 1.0 Public domain dedication).

## Discussion

Comparing *π*_clinic_=47.4% at the “Edu”=“Hi” with *π*_clinic_=42.74%=“Edu”=“Uni”, it can be concluded that people with lower levels of education (high school or less) are more likely to go to clinics than those with a higher education (university or above). Also, a change of *π*_clinic_ from 43.7% to 51.6% when “PopularInfo” runs from 1 to 5 points proves that effective communication will increase the likelihood of people going to clinics when finding illness symptoms. Similarly, *π _clinic_* also increases if physicians’ responsiveness is rated at a high level. Moreover, it can be seen that the older people are, the higher the probability they prioritise visiting clinics ( Table 4a).

**Table 4.** Distribution of conditional probabilities.

Probabilities of “Clinic” vary according to “Age”, “PopularInfo” and “Respon” (4a)					
Condition	“Edu”=“Hi”, “Age”=30, “PopularInfo”=2.8				
“Respon”	1	2	3	4	5
*π*_clinic_	0.422	0.445	0.467	0.485	0.501
Condition	“Edu”=“Hi”, “Age”=30, “Respon”=3.38				
“PopularInfo”	1	2	3	4	5
*π*_clinic_	0.437	0.458	0.478	0.497	0.516
Conditions	“Edu”=“Hi”, “PopularInfo”=2.8, “Respon”=3.38				
“Age”	10	30	50	70	90
*π _clinic_*	0.275	0.474	0.669	0.810	0.894
Probabilities of “AfterIT”=“yes” vary according to “Age” and “PopularInfo” (4b)					
Condition	“UseIT”=“yes”, “PopularInfo”=2.8				
“Age”	10	30	50	70	90
*π _yes_*	0.703	0.688	0.667	0.635	0.591
Condition	“UseIT”=“yes”, “Age”=30				
“PopularInfo”	1	2	3	4	5
*π _yes_*	0.674	0.682	0.690	0.696	0.702
Probabilities of “QualExam” vary according to “SuffInfo” and “PopularInfo” (4c)					
Condition	“PopularInfo”=2.8				
“SuffInfo”	1	2	3	4	5
*π _hi_*	0.278	0.312	0.344	0.374	0.403
*π _low_*	0.079	0.042	0.022	0.011	0.006
Condition	“SuffInfo”=3.01				
“PopularInfo”	1	2	3	4	5
*π _hi_*	0.267	0.308	0.354	0.402	0.451
*π _low_*	0.024	0.023	0.021	0.020	0.018

From the two equations ( Eq.3) and ( Eq.4), it can be observed that the absolute value of the coefficient corresponding to the variable “UseIT” is the largest, with *β*_3_=-2.558 ( *P* < 0.001) at ( Eq.3) and *β*_2_=2.022 ( *P* < 0.001) at ( Eq.4). It means that the increase or decrease of the probability of attending GHE after using IT apps will bear the greatest impact from the readiness or hesitation toward using IT health apps. In addition, Table 4b shows that the likelihood of attending GHE after using IT apps decreases as age increases. In contrast, this figure increases when health communication becomes increasingly popular.

Regarding assessment of the quality of healthcare services, the probability of a high score is larger than a low score in all conditions, especially when the efficiency of communication and the sufficiency of information reach the highest point (5 points), the probability that healthcare quality is assessed highly is largest (π _hi_ > 40%). Therefore, it can be stated that the more widely and adequately information is disseminated, the more probable people will feel positive about healthcare quality ( Table 4c).

**Figure 1.**
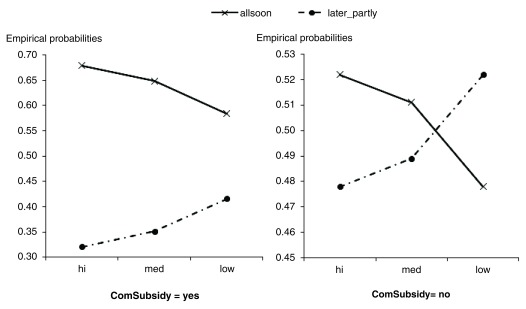
Probability of using a cash subsidy for GHE of a person expressing hesitation, due to its non-urgency and unimportance. The figure represents trends of changing probabilities using funds available for GHEs, which control for the provision of community cash support. With community subsidies, respondents showed a stronger propensity to quickly use up the funds for GHEs.

It can be seen that the regression coefficient *β*_1_ of variable “NotImp” in ( Eq.7) is negative and is positive in ( Eq.8). Therefore, those who are hesitant, due to considering GHEs as not urgent and important, are less likely to make use of the total subsidy in the near future. The influence of “ComSubsidy” and “AffCost” are clarified through the analyses of Figure 1.

Firstly, it can be seen that the probability line of “using all the money soon” (“allsoon”) in both the charts in Figure 1 have downward trends when moving from point “hi” to point “low” of “AffCost”, whereas the opposite trend occurs for the “later_partly” line. This means that the probability of using all the money soon reduces when people are willing to pay a high cost for a GHE. Moreover, ( Eq.7) and ( Eq.8) also imply that acceptable costs have the strongest impact on the use of provided money for GHEs.

Furthermore, the probability line of “allsoon” ranges from over 55% to nearly 70% in Figure 1 (left panel) and from over 47% to nearly 53% on the right panel. Therefore, participants tend to take all the money for an early GHE if they receive a subsidy from the community or government.

Finally, the two probability lines in Figure 1 (left panel) lie separately, while those in the right panel intersect with one another. This proves that when a person demonstrates a willingness toward GHEs, due to a community subsidy, then they tend to give priority to GHEs.

## Conclusion

The analyses in the present study helps to provide some valuable conclusions as follows:

IT apps increase the likelihood of GHE participation, as 83% of participants said they might or would definitely visit a doctor if the apps reveal health problems or illness symptoms. The remainder expressed doubts on the reliability of the apps. This usually occurred in older people; nearly ¾ of people aged above 50 years did not completely trust the quality of these mobile apps.

Educational attainment is also a strong influence on the decision of GHE participation (with *β*_2_=0.712 ( *P*<0.001) at ( Eq.1) and *β*_2_=0.578 ( *P*<0.001), following ( Eq.2)). The preventive medicine or subclinical tests applied in GHE require inquiry and a certain amount of knowledge, which is limited for the people with a lower level of education. In this case, the clinical methods appear more effective. These people are eager to get direct advice from relatives, friends or doctors, while only about 18% of participants preferred self-study.

By contrast, effective health communications helped participants to have enough information and a thus formed a more trustworthy base, forming standards of comparison instead of purely emotional and personal conclusions, so that the evaluation tends to be improved and more objective. The proof is that nearly 70% of respondents rated the quality of healthcare services highly if they rated the sufficiency and coverage of information highly. Moreover, ITs also reduce the expensiveness of information ^36^. However, health communications in Vietnam are still defective, especially as they are less widespread (assessment of efficacy: 2.8 out of 5 points; Table 2). Therefore, people expect a better coverage of health information.

Apart from ICTs, the community/government subsidy is also one measure that promotes GHEs. People tend to attend early GHEs when they receive cash subsidies (58.4 – 79.1%). However, about 52% of participants do not appreciate the importance of regular check-ups ( Table 1). This may be due to limited finance (accounting for 60.8%), but might also be because they feel GHEs are not really necessary; therefore, they could use the subsidy for other improper purposes (accounting for 37.81%). For that reason, the authorities/communities need support in a reasonable manner in order to further promote the public’s readiness toward GHEs for their family and themselves.

Also, it cannot be denied that the quality of healthcare services in clinics and hospitals, particularly the responsiveness of nurses and doctors, remains low. With an average of 3.38 out of 5 points, responsiveness is rated lowest among the five elements included, whereas the empirical average score for quality of medical services is only at a medium level (3.55 out of 5 points). This somewhat reduces peoples’ desire to go to hospitals to check their health. Therefore, it is definitely necessary to improve the quality of medical services in Vietnam, especially public hospitals, since people tend to be more satisfied with private hospitals ^31^.

## Data availability

The data referenced by this article are under copyright with the following copyright statement: Copyright: © 2016 Vuong QH

Data associated with the article are available under the terms of the Creative Commons Zero "No rights reserved" data waiver (CC0 1.0 Public domain dedication). 

**Dataset 1: Raw data gathered from the survey, doi,** 10.5256/f1000research.10508.d147548 ^42^. The data table used for providing descriptive statistics and preparing data subsets for statistical analysis (see also Supplementary Table 1).

## Supplementary material

**Supplementary File 1:** The survey questionnaire is provided in full (in Vietnamese).

.

**Supplementary File 2:** The survey questionnaire is provided in full (in English).

.

**Supplementary File 3: Estimations in R.** These data files are available for verification and re-confirmation of the results found by the present study.

.

**Supplementary Table 1: Contingency table for estimations.** Counts for relevant factors involved in statistical analysis.

## References

[1] Cherrington A Corbie-Smith G Pathman DE: Do adults who believe in periodic health examinations receive more clinical preventive services? *Prev Med.* 2007;45(4):282–289. 10.1016/j.ypmed.2007.05.016 17692368 PMC3757124

[2] Burton LC Steinwachs DM German PS: Preventive services for the elderly: would coverage affect utilization and costs under Medicare? *Am J Public Health.* 1995;85(3):387–391. 10.2105/AJPH.85.3.387 7892924 PMC1614868

[3] Finkelstein MM: Preventive screening. What factors influence testing? *Can Fam Physician.* 2002;48:1494–1501. 12371308 PMC2214098

[4] Nakanishi N Tatara K Fujiwara H: Do preventive health services reduce eventual demand for medical care? *Soc Sci Med.* 1996;43(6):999–1005. 10.1016/0277-9536(96)00016-0 8888469

[5] Garritty C El Emam K: Who’s using PDAs? Estimates of PDA use by health care providers: a systematic review of surveys. *J Med Internet Res.* 2006;8(2):e7. 10.2196/jmir.8.2.e7 16867970 PMC1550702

[6] Baldwin LP Low PH Picton C: The use of mobile devices for information sharing in a technology-supported model of care in A&E. *Int J Electron Healthc.* 2007;3(1):90–106. 10.1504/IJEH.2007.011482 18048263

[7] Ozdalga E Ozdalga A Ahuja N: The smartphone in medicine: a review of current and potential use among physicians and students. *J Med Internet Res.* 2012:14(5):e128. 10.2196/jmir.1994 23017375 PMC3510747

[8] Koehler N Vujovic O McMenamin C: Healthcare professionals’ use of mobile phones and the internet in clinical practice. *JMTM.* 2013;2:3–13. 10.7309/jmtm.2.1.2

[9] West D: How mobile devices are transforming healthcare. *Issues in Tech Innovation.* 2012;18:1–14. Reference Source

[10] Hamilton AD Brady RR: Medical professional involvement in smartphone ‘apps’ in dermatology. *Br J Dermatol.* 2012;167(1):220–221. 10.1111/j.1365-2133.2012.10844.x 22283748

[11] Abboudi H Amin K: Smartphone applications for the urology trainee. *BJU Int.* 2011;108(9):1371–1373. 10.1111/j.1464-410X.2010.10640.x 22023058

[12] Kaplan WA: Can the ubiquitous power of mobile phones be used to improve health outcomes in developing countries? *Global Health.* 2006;2:9. 10.1186/1744-8603-2-9 16719925 PMC1524730

[13] Martinez AW Phillips ST Carrilho E: Simple telemedicine for developing regions: camera phones and paper-based microfluidic devices for real-time, off-site diagnosis. *Anal Chem.* 2008;80(10):3699–3707. 10.1021/ac800112r 18407617 PMC3761971

[14] Fraser HS Jazayeri D Nevil P: An information system and medical record to support HIV treatment in rural Haiti. *BMJ.* 2004;329(7475):1142–1146. 10.1136/bmj.329.7475.1142 15539669 PMC527691

[15] Ventola CL: Mobile devices and apps for health care professionals: uses and benefits. *P T.* 2014;39(5):356–364. 24883008 PMC4029126

[16] Buijink AW Visser BJ Marshall L: Medical apps for smartphones: lack of evidence undermines quality and safety. *Evid Based Med.* 2013;18(3):90–92. 10.1136/eb-2012-100885 22923708

[17] Rosser BA Eccleston C: Smartphone applications for pain management. *J Telemed Telecare.* 2011;17(6):308–312. 10.1258/jtt.2011.101102 21844177

[18] Visvanathan A Hamilton A Brady RR: Smartphone apps in microbiology--is better regulation required? *Clin Microbiol Infect.* 2012;18(7):E218–220. 10.1111/j.1469-0691.2012.03892.x 22563840

[19] Gandjour A Lauterbach KW: Preventive care and the prospect of cost savings. *Eur J Health Econ.* 2006;3:1–2.

[20] Fletcher RH: Review: Periodic health examination increases delivery of some clinical preventive services and reduces patient worry. *Evid Based Med.* 2007;12(4):118. 10.1136/ebm.12.4.118 17885166

[21] Merenstein D Daumit GL Powe NR: Use and costs of nonrecommended tests during routine preventive health exams. *Am J Prev Med.* 2006;30(6):521–527. 10.1016/j.amepre.2006.02.003 16704947

[22] Gurchiek K: Health contributions tied to workers’ pay. *HR Magazine.* 2005;50(2):28–32. Reference Source

[23] Vuong QH: Be rich or don’t be sick: estimating Vietnamese patients’ risk of falling into destitution. *Springerplus.* 2015;4:529. 10.1186/s40064-015-1279-x 26413435 PMC4577521

[24] Lim PC Tang NK: A study of patients' expectations and satisfaction in Singapore hospitals. *Int J Health Care Qual Assur Inc Leadersh Health Serv.* 2000;13(6–7):290–299. 10.1108/09526860010378735 11484647

[25] Khan MH Hassan R Anwar S: Patient satisfaction with nursing care. *RMJ.* 2007;32(1):28–30. Reference Source

[26] Bleich SN Ozaltin E Murray CK: How does satisfaction with the health-care system relate to patient experience? *Bull World Health Organ.* 2009;87(4):271–278. 19551235 10.2471/BLT.07.050401 PMC2672587

[27] Valentine N Darby C Bonsel GJ: Which aspects of non-clinical quality of care are most important? Results from WHO's general population surveys of “health systems responsiveness” in 41 countries. *Soc Sci Med.* 2008;66(9):1939–1950. 10.1016/j.socscimed.2007.12.002 18313822

[28] Epner JE Levenberg PB Schoeny ME: Primary care providers' responsiveness to health-risk behaviors reported by adolescent patients. *Arch Pediatr Adolesc Med.* 1998;152(8):774–780. 9701137 10.1001/archpedi.152.8.774

[29] Andaleeb SS: Service quality perceptions and patient satisfaction: a study of hospitals in a developing country. *Soc Sci Med.* 2001;52(9):1359–1370. 10.1016/S0277-9536(00)00235-5 11286361

[30] González-Valentín A Padín-López S de Ramón-Garrido E: Patient satisfaction with nursing care in a regional university hospital in southern Spain. *J Nurs Care Qual.* 2005;20(1):63–72. 15686078 10.1097/00001786-200501000-00011

[31] Uzun Ö: Evaluation of satisfaction with nursing care of patients hospitalized in surgical clinics of different hospitals. *IJCS.* 2015;8(1):19–24. Reference Source

[32] Coulter A Jenkinson C: European patients' views on the responsiveness of health systems and healthcare providers. *Eur J Public Health.* 2005;15(4):355–360. 10.1093/eurpub/cki004 15975955

[33] Keller PA Lehmann DR: Designing effective health communications: a meta-analysis. *JPP&M.* 2008;27(2):117–130. 10.1509/jppm.27.2.117

[34] Block LG Keller PA: Effects of self-efficacy and vividness on the persuasiveness of health communications. *J Consum Psychol.* 1997;6(1):31–54. 10.1207/s15327663jcp0601_02

[35] Sutton SM Eisner EJ Burklow J: Health communications to older Americans as a special population. The National Cancer Institute's consumer-based approach. *Cancer.* 1994;74(7 Suppl):2194–2199. 10.1002/1097-0142(19941001)74:7+<2194::AID-CNCR2820741734>3.0.CO;2-E 8087791

[36] Vuong QH: Information expensiveness perceived by Vietnamese patients with respect to healthcare provider's choice. *Acta Inform Med.* 2016;24(5):280–283. 10.5455/aim.2016.24.360-363 PMC5203750 28077894

[37] Kreps GL: Communication and health education. In *Communication and health: Systems and Applications* (ed. Eileen B, & Lewis D). Routledge,1990;187–203. Reference Source

[38] Kreps GL Kunimoto EN: Effective communication in multicultural health care settings. Sage Publications,1994 10.4135/9781483326344

[39] Patrick K Intille SS Zabinski MF: An ecological framework for cancer communication: implications for research. *J Med Internet Res.* 2005;7(3):e23. 10.2196/jmir.7.3.e23 15998614 PMC1550654

[40] Vuong QH Nguyen TK: Vietnamese patients' choice of healthcare provider: in search of quality information. *Int J Behav Healthcare Res.* 2015;5(3/4):184–212. 10.1504/IJBHR.2015.077678

[41] Kanavos P: The rising burden of cancer in the developing world. *Ann Oncol.* 2006;17(Suppl 8):viii15–viii23. 10.1093/annonc/mdl983 16801335

[42] Vuong QH: Dataset 1 in: Health communication, information technology and the public’s attitude toward periodic general health examinations. *F1000Research.* 2016 Data Source 10.12688/f1000research.10508.1 PMC5247783 28163904

