# Supplementary material for: Health communication, information technology and the public’s attitude toward periodic general health examinations
Source: F1000Res. 2016 Dec 30;5:2935. [Version 1] doi: 10.12688/f1000research.10508.1 (PMC5247783; doi:10.12688/f1000research.10508.1)
Supplement: Supplementary file 2 [file f1000research-5-11326-s0001.tgz › 302bbe45-0342-49ba-b107-4e4fef0570c9.docx]

**Khảo sát xu hướng khám sức khỏe định kỳ**

| *Xin chân thành cảm ơn quý vị tham gia khảo sát y tế cộng đồng.*  ** Nghiên cứu nhằm tìm hiểu xu hướng và các nhân tố động viên/hạn chế cộng đồng sử dụng dịch vụ khám sức khỏe định kỳ.*  ** Đơn vị điều phối: văn phòng Vuong & Associates.*  ** Khảo sát tuân thủ Tuyên bố chuẩn mực đạo đức kèm ở cuối bảng hỏi.*  ** Kết quả sẽ được công bố rộng rãi nhằm phục vụ chính sách y tế công trong tương lai.* |
| --- |

| Phiếu số:  .................................................................................. | Người trả lời:  .................................................................................. |
| --- | --- |
| Ngày ghi dữ liệu:  ............/............/............. | Tuổi:  ............................................................... |
| Giới tính: □Nam □Nữ | Công việc: □Ổn định □Không ổn định □Học sinh/sinh viên □Hưu trí □Nội trợ □Khác |
| Tình trạng hôn nhân:  □Đã kết hôn □Chưa □ Khác | Trình độ học vấn:  □THCS □THPT □Đại học □Sau ĐH |
| Chiều cao (cm): ....................................................... | Nơi cư trú: ................................................................ |
| Cân nặng (kg): ........................................................ | Chỉ số BMI: ................................ □Không biết |
| Bảo hiểm y tế: □Có □Không | Chữ ký:  .................................................................... |

* * *

(*Lưu ý: Với mỗi câu hỏi dưới đây, đánh dấu chọn chỉ một ô duy nhất*)

**1. Thói quen khám bệnh:**

| 1.1. Lần khám bệnh gần nhất | □<12 tháng | □12-24 tháng | □>24 tháng | □Không nhớ |
| --- | --- | --- | --- | --- |
| 1.2. Lần khám tổng quát gần nhất | □<12 tháng | □12-24 tháng | □>24 tháng | □Không nhớ |
| 1.3. Lý do khám gần nhất |  |  |  |  |

□Thông tin bệnh / dịch

□Có dấu hiệu sức khỏe gây lo lắng

□Theo yêu cầu cơ quan / cộng đồng / bảo hiểm ...

□Tự nguyện (bao gồm sự nhất trí trong gia đình) dù không có vấn đề sức khỏe

**2. Tâm lý khám tổng quát định kỳ:**

| 2.1. Lý do ngại khám: | |  |  | |  | |  |
| --- | --- | --- | --- | --- | --- | --- | --- |
| 2.1.1. Tốn thời gian: | | | □Có | | □Không | |  |
| 2.1.2. Tốn kém tiền bạc: | | | □Có | | □Không | |  |
| 2.1.3. Sợ phát hiện ra bệnh: | | | □Có | | □Không | |  |
| 2.1.4. Kém tin tưởng vào chất lượng: | | | □Có | | □Không | |  |
| 2.1.5. Chưa cấp bách / không quan trọng: | | | □Có | | □Không | |  |
| 2.2. Lý do sẵn sàng khám: | | | | | | |  |
|  | | | | | | |  |
| 2.2.1. Sức khỏe ưu tiên hàng đầu: | | | □Đúng | | □Không | |  |
| 2.2.2. Được trợ cấp của cơ quan/cộng đồng: | | | □Có | | □Không | |  |
| 2.2.3. Thói quen của gia đình/cơ quan: | | | □Đúng | | □Không | |  |
| 2.2.4. Thường xuyên theo dõi thông tin sức khỏe: | | | □Có | | □Không | |  |

**3. Tình hình sức khỏe cá nhân/gia đình:**

| 3.1. Đã/đang điều trị dài ngày: | □Đúng | □Chưa bao giờ |
| --- | --- | --- |
| 3.2. Có bạn/người thân đã/đang điều trị: | □Đúng | □Chưa bao giờ |
| 3.3. Tương đối tốt/ổn định: | □Đúng | □Không |

**4. Dụng cụ/kiến thức/thực hành y tế trong gia đình:**

| 4.1. Có tủ thuốc với một số thuốc/dụng cụ phổ thông: | □Có | □Không |
| --- | --- | --- |
| 4.2. Có kỹ năng dùng một số dụng cụ y tế cơ bản: | □Đúng | □Không |
| 4.3. Đã từng chăm sóc người thân bị ốm: | □Đúng | □Chưa |
| 4.4. Kiểm tra y tế đơn giản thường xuyên trong gia đình (đo huyết áp, cân nặng, thị lực, theo dõi dấu hiệu...): | □Đúng | □Không |

**5. Nếu được cấp tiền mặt để khám sức khỏe định kỳ, quý vị sẽ:**

| □Sử dụng hết và khám sớm |
| --- |
| □Sử dụng một phần, và tiết kiệm phần còn lại: |
| □Nhận số tiền và khám vào dịp khác thuận tiện: |

**6. Đánh giá cá nhân về chất lượng dịch vụ khám sức khỏe định kỳ:**

(*Riêng câu hỏi từ 6.1 đến 6.5, cho điểm từ 1 đến 5; 1 là kém nhất, 5 là cao nhất*)

6.1. Điểm về hình thức, chuẩn mực thiết bị, đội ngũ (Tangibles): ...... điểm

6.2. Năng lực đáp ứng tiêu chuẩn khám đúng yêu cầu (Reliability): ...... điểm

6.3. Tính sẵn sàng và tránh lãng phí thời gian (Responsiveness): ...... điểm

6.4. Kiến thức/khả năng tạo độ tin cậy chuyên môn (Assurance): ...... điểm

6.5. Mức độ chu đáo, trách nhiệm và ân cần chăm sóc (Empathy): ...... điểm

**7. Khi có dấu hiệu sức khỏe cần quan tâm, lựa chọn kiểm tra đầu tiên là:**

| □Tới phòng khám | □Hỏi gia đình / người quen | □Tự tìm hiểu |
| --- | --- | --- |

**8. Cảm nhận về tình hình sức khỏe cộng đồng của quý vị:**

| □Tốt | □Cơ bản tốt, dù có vấn đề | □Không tốt, nan giải | □Không biết |
| --- | --- | --- | --- |

**9. Tần suất khám tổng quát nào phù hợp với quý vị?**

□6 tháng/lần □12 tháng □18 tháng □>18 tháng

**10. Chi phí khám tổng quát (nếu phải tự trả) nào dưới đây chấp nhận được?**

□<1 triệu đồng/lần □1-2 triệu □>2 triệu

**11. Quý vị có sẵn sàng sử dụng ứng dụng công nghệ thông tin được đánh giá đáng tin cậy (vd. Smartphone apps) để giúp nhận biết dấu hiệu sức khỏe cần kiểm tra?**

□Có □Có thể □Không

**12. Nếu ứng dụng CNTT chỉ ra nhu cầu khám một số vấn đề, quý vị sẽ khám tổng quát?**

□Có □Có thể □Không

**13. Đánh giá về thông tin/tuyên truyền về khám sức khỏe tổng quát theo đánh giá của quý vị?**

(*Các câu hỏi từ 13.1 đến 13.4, cho điểm từ 1 đến 5; 1 là kém nhất, 5 là cao nhất*)

13.1. Tính đầy đủ: ...... điểm

13.2. Mức độ hấp dẫn (gây chú ý): ...... điểm

13.3. Tính nhấn mạnh (có trọng điểm): ...... điểm

13.4. Tính phổ biến (đều đặn): ...... điểm

**14. Thời gian vận động/thể dục và thể thao bao lâu là phù hợp để đảm bảo rèn luyện sức khỏe?**

................ phút / tuần □Không biết

**15. Quý vị tự đánh giá thời gian mình dành cho vận động/thể dục, thể thao so với mức cần thiết?**

□Rất đủ □Tương đối đủ □Có, nhưng ít □Không đáng kể

**Tuyên bố về chuẩn mực đạo đức nghiên cứu**

| Chúng tôi tự nguyện tuyên bố và cam kết tuân thủ các chuẩn mực về thu thập, lưu trữ, xử lý và phân tích dữ liệu, dựa trên các tiêu chuẩn khoa học cao nhất về:   - Tính trung thực: Thông tin chính xác về căn cứ, bản chất, mục đích và kết cục nghiên cứu. - Sự đồng ý: Người tham gia trả lời hiểu rõ và đủ về câu hỏi nghiên cứu và đủ thông tin để lựa chọn có tham gia trả lời hay không. - Bảo vệ người dễ tổn thương: Chúng tôi hiểu rõ bổn phận đảm bảo quyền lợi của những người dễ bị tổn thương và hạn chế rủi ro ở mức thấp nhất. - Bảo toàn riêng tư: Người trả lời có quyền từ chối trả lời một phần, toàn bộ, cũng như quyền rút lui khỏi việc khảo sát tại bất kỳ thời điểm nào họ muốn. - Bảo mật: Thông tin được bảo mật và chỉ sử dụng cho nghiên cứu khoa học, không được cung cấp cho bên thứ ba mà để lộ danh tính người tham gia. - Tối thiểu hóa rủi ro: Hạn chế tới mức thấp nhất bất kỳ rủi ro nào liên quan tới người trả lời. - Phổ biến kết quả: Kết quả nghiên cứu sẽ được công bố (không nêu danh tính người tham gia) dựa trên nguyên tắc minh bạch, được thẩm định và bình duyệt khoa học. Khi điều kiện cho phép, dữ liệu sau khi loại bỏ danh tính sẽ được lưu trữ phụng sự nghiên cứu khoa học. |
| --- |

| *Người lập phiếu* | *Kiểm tra* |
| --- | --- |
| ................................................  Ngày: ...................................... | Ngày: ...................................... |
| *Phụ trách nhóm dữ liệu* | *Nghiên cứu viên chính* |
| Ngày: ...................................... | Ngày: ...................................... |

[*Ngày hoàn thiện mẫu phiếu điều tra*: 18/9/2016]
