# Supplementary material for: Health communication, information technology and the public’s attitude toward periodic general health examinations
Source: F1000Res. 2016 Dec 30;5:2935. [Version 1] doi: 10.12688/f1000research.10508.1 (PMC5247783; doi:10.12688/f1000research.10508.1)
Supplement: Supplementary file 3 [file f1000research-5-11326-s0002.tgz › 08204d4f-dde9-4fe3-9ac9-34fcc84eb2fb.docx]

**Survey on the trend of periodic general health examinations**

| *We sincerely thank you for participating in this community health survey.*  ** This survey is to understand the trends and factors that encourage/discourage the community to periodically use general health examinations.*  ** The survey team: Vuong & Associates.*  ** The survey team observes and abide by regulations and stipulations provided by the Declaration of Ethical Standards, which are enclosed to this questionnaire.*  ** Results will be published for free use of the public, serving the future public health policy making.* |
| --- |

| Record No.:  .................................................................................. | Name of respondent:  .................................................................................. |
| --- | --- |
| Date of record:  ............/............/............. | Age:  ............................................................... |
| Gender: □Male □Female | Job status: □Employed □Unemployed □Student □Housework □Retired □Others |
| Marital status:  □Married □Single □ Others | Education:  □Junior high □High school □College □Graduate |
| Height (cm): ....................................................... | Place of living: ................................................................ |
| Weight (kg): ........................................................ | Your BMI: ................................ □I don't know |
| Medical insurance: □Yes □No | Signature of respondent:  .................................................................... |

* * *

(*Note: For each of the following questions, select only one choice*)

**1. Habit of attending physical examination:**

| 1.1. Latest health examination | □<12 months | | □12-24 m | □>24 m | □Don't remember |
| --- | --- | --- | --- | --- | --- |
| 1.2. Latest GHE | □<12 m | | □12-24 m | □>24 m | □Don't remember |
| 1.3. Reason for the latest health check-up | |  |  |  |  |

□Information about disease outbreaks

□Some worrying symptoms appearing

□At request of the employer and/or insurance agents / community...

□Voluntary, including advice from the family although without signs of illness

**2. Psychological issues:**

| 2.1. Reasons why you are discouraged from attending GHE: | | |  |  |  | |  |
| --- | --- | --- | --- | --- | --- | --- | --- |
| 2.1.1. Time consuming: | □True | | | | □Not true | |  |
| 2.1.2. Financially costly: | □True | | | | □Not true | |  |
| 2.1.3. Afraid of diseases/illness: | □True | | | | □Not true | |  |
| 2.1.4. Low trust in GHE quality: | □True | | | | □Not true | |  |
| 2.1.5. Not critical / not imperative: | □True | | | | □Not true | |  |
| 2.2. Reasons why you are encouraged to attend GHE: | | | | | | |  |
|  | | | | | | |  |
| 2.2.1. Health is the first priority in life: | □True | | | | □Not true | |  |
| 2.2.2. Subsidy from employer or community: | □True | | | | □Not true | |  |
| 2.2.3. Long-standing habits of the family/community: | □True | | | | □Not true | |  |
| 2.2.4. Following news about health frequently: | □True | | | | □Not true | |  |

**3. Personal and family health status:**

| 3.1. Under long-term treatment now or in the past: | □Yes | □No |
| --- | --- | --- |
| 3.2. Having close friends or relatives with serious medical treatments: | □Yes | □No |
| 3.3. In good health now: | □Yes | □No |

**4. Medical equipment/tools and related knowledge about basic medical practices in-house:**

| 4.1. Have first-aid kit and several common medicine in house: | □Yes | □No |
| --- | --- | --- |
| 4.2. Have skills about using basic medical tools and materials: | □Yes | □No |
| 4.3. Have experience of taking care of ill relatives: | □Yes | □No |
| 4.4. Practice simple tests at home (e.g. blood pressure, weight, checking simple symptoms...): | □Yes | □No |

**5. If provided with cash subsidy for GHE, you would:**

| □Use up the funds, quickly |
| --- |
| □Use part of the funds, and save the remaining |
| □Take the money and attend GHE at a later date |

**6. Your assessment of periodic GHE quality:**

(*For questions from 6.1 to 6.5, give lowest score of 1 and highest 5*)

6.1. Score about appearance, professionalism, staff (Tangibles): ...... point

6.2. Score about reliable GHE services (Reliability): ...... point

6.3. Readiness and time saving for examinees (Responsiveness): ...... point

6.4. Knowledge and trust-building by GHE professionals (Assurance): ...... points

6.5. Care taking, responsibility of staff and service provider (Empathy): ...... points

**7. When you worry about health symptoms, your first choice is:**

| □Clinical service | □Family / friends | □Self-study |
| --- | --- | --- |

**8. Your view about the current public health situation now:**

| □Generally good | □Some problems, but not critical | □Not good at all, serious issues remain | □Don't care |
| --- | --- | --- | --- |

**9. How frequent would you like a GHE to be?**

□6 months □12 m □18 m □>18 m

**10. What cost is acceptable (if you have to pay it yourself) for a GHE?**

□<VND 1 mn/GHE □VND 1-2 mn □>VND 2 mn

**11. Are you willing to use IT equipment and Smartphone apps to learn about your own health status?**

□Yes □Maybe □No

**12. If the IT equipment reveals health issues, would you attend a GHE?**

□Yes □Maybe □No

**13. Your assessment about health and healthcare communication in Vietnam?**

(*For questions from 13.1 to 13.4, give lowest score of 1 and highest 5*)

13.1. Completeness: ...... points

13.2. Attention: ...... pts

13.3. Stress: ...... pts

13.4. Universal: ...... pts

**14. How much time for physical activity/training is enough for you?**

................ minutes / week □I don't know

**15. Do you think you spend enough time doing physical activities and training in respect of health maintenance?**

□Very healthy □Basically enough □Some, but still inadequate □Too little

**Declaration on ethical standards of this medical survey**

| We ensure the basic principles of ethical research as stated below:   - Beneficence: As a researcher we strive to ensure that my work makes a positive contribution to the welfare of those affected by it. - Non-malfeasance: we endeavour to ensure that the research work does not cause harm to any sectors of society and, in particular, to participants. - Justice: The benefits and risks associated with this study should be well assessed in advance and both should be equitably distributed throughout society. - Autonomy of subjects: The research respects and protects the rights and dignity of participants.   As the research involves primary data collection, in the form of interview surveys, we have been fully compliant with all legal requirements regarding the collection, storage, handling, processing and analysis of data. We guarantee that we have conformed to the highest standards of:   - Veracity: Participants in sample surveys and data collection exercises are given full and accurate information regarding issues such as the background, nature, purpose, and outputs of the research. - Informed Consent: Participants in sample surveys and related data collection exercises were given sufficient details on the research in question as to allow them to make an informed decision to participate or otherwise in a research study. - Protection of Vulnerable Groups: we are particularly conscious of our obligations to safeguard the interests of vulnerable or potentially ‘at risk’ groups who may be involved in the research. - Privacy: Participants in data collection exercises have the right not only to agree to participate in the research but also to decide to withdraw from the research at any time. - Confidentiality: The information provided by participants is treated as confidential and used for research purposes only. Micro-level information will not be disclosed in any fashion to third parties, which would allow it to be associated with an identifiable individual. - Minimising Risk: Participants in the research will not be put under undue or unnecessary risk as a result of their participation. - Research Outputs: we are committed to putting the results of this research into the public domain (always on an anonymous basis) with a view to transparency, scrutiny and peer review. Where feasible, we are committed to depositing anonymous primary data collected in the course of research into a publicly accessible data archive. |
| --- |

| *Undertaker* | *Checked by:* |
| --- | --- |
| ................................................  Date: ...................................... | Date: ...................................... |
| *Supervisor of the data team* | *The researcher (Quan-Hoang Vuong)* |
| Date: ...................................... | Date: ...................................... |

[*Date of this questionnaire form*: 18/9/2016]
