# Supplementary material for: Health communication, information technology and the public’s attitude toward periodic general health examinations
Source: F1000Res. 2016 Dec 30;5:2935. [Version 1] doi: 10.12688/f1000research.10508.1 (PMC5247783; doi:10.12688/f1000research.10508.1)
Supplement: Supplementary file 4 [file f1000research-5-11326-s0003.tgz › 941ba476-9421-4b0c-bf95-219cc187170c.pdf]

Regression with response variable "StChoice" and predictors "Edu", "Age", "Respon", "PopularInfo"

```
> med4.3=read.csv("D:/V&A/Med4/Med4.3/11102016Med4.3.csv",header=T)

> attach(med4.3)

> contrasts(med4.3$Edu)=contr.treatment(levels(med4.3$Edu),base=2)

> fit.mdl1=vglm(formula =
StChoice~Edu+Age+Respon+PopularInfo,data=med4.3,family=multinomial)

> summary(fit.mdl1)
```

```
Call:
vglm(formula = StChoice ~ Edu + Age + Respon + PopularInfo, family = multinomial,
    data = med4.3)

Pearson residuals:
                      Min      1Q  Median    3Q   Max
log(mu[,1]/mu[,3]) -1.892 -0.7919 -0.2987 1.137 3.832
log(mu[,2]/mu[,3]) -2.503 -0.9667 -0.3693 1.045 1.620

Coefficients:
               Estimate Std. Error z value Pr(>|z|)
(Intercept):1  1.004403   0.276257   3.636 0.000277 ***
(Intercept):2 -0.673077   0.253440  -2.656 0.007913 **
EduHi:1        0.711541   0.146893   4.844 1.27e-06 ***
EduHi:2        0.578150   0.136783   4.227 2.37e-05 ***
Age:1         -0.024962   0.007261  -3.438 0.000587 ***
Age:2          0.026068   0.005962   4.372 1.23e-05 ***
Respon:1      -0.224826   0.047743  -4.709 2.49e-06 ***
Respon:2      -0.066916   0.044538  -1.502 0.132984
PopularInfo:1  0.122978   0.051283   2.398 0.016484 *
PopularInfo:2  0.158654   0.047304   3.354 0.000797 ***
---
Signif. codes:  0 '***' 0.001 '**' 0.01 '*' 0.05 '.' 0.1 ' ' 1

Number of linear predictors:  2

Names of linear predictors: log(mu[,1]/mu[,3]), log(mu[,2]/mu[,3])

Dispersion Parameter for multinomial family:   1

Residual deviance: 4304.026 on 4126 degrees of freedom

Log-likelihood: -2152.013 on 4126 degrees of freedom

Number of iterations: 4

Reference group is level  3  of the response
```

Regression with response variable "AfterIT" and predictors "Age", "UseIT", "PopularInfo"

```
> attach(med4.3)

> fit.mdl2=vglm(formula =

AfterIT~Age+UseIT+PopularInfo,data=med4.3,family=multinomial)

> summary(fit.mdl2)
```

```
Call:
vglm(formula = AfterIT ~ Age + UseIT + PopularInfo, family = multinomial,
    data = med4.3)

Pearson residuals:
                      Min      1Q   Median       3Q   Max
log(mu[,1]/mu[,3]) -2.151 -0.6222 -0.22053  0.50718 2.664
log(mu[,2]/mu[,3]) -2.224 -0.2646 -0.09855 -0.05573 6.793

Coefficients:
               Estimate Std. Error z value Pr(>|z|)
(Intercept):1  1.623530   0.237585   6.833 8.29e-12 ***
(Intercept):2 -1.290375   0.340883  -3.785 0.000153 ***
Age:1          0.001001   0.006055   0.165 0.868723
Age:2          0.026326   0.007586   3.470 0.000520 ***
UseITno:1     -1.744164   0.177694  -9.816  < 2e-16 ***
UseITno:2      2.022381   0.222354   9.095  < 2e-16 ***
UseITyes:1    -2.557619   0.128717 -19.870  < 2e-16 ***
UseITyes:2    -1.773842   0.258607  -6.859 6.92e-12 ***
PopularInfo:1 -0.008027   0.047611  -0.169 0.866117
PopularInfo:2 -0.210212   0.067932  -3.094 0.001972 **
---
Signif. codes:  0 '***' 0.001 '**' 0.01 '*' 0.05 '.' 0.1 ' ' 1

Number of linear predictors:  2

Names of linear predictors: log(mu[,1]/mu[,3]), log(mu[,2]/mu[,3])

Dispersion Parameter for multinomial family:   1

Residual deviance: 3081.751 on 4126 degrees of freedom

Log-likelihood: -1540.876 on 4126 degrees of freedom

Number of iterations: 6

Reference group is level  3  of the response
```

Regression with response variable “QualExam” and predictors “SufffInfo”, “PopularInfo”

```
> attach(med4.3)

> fit.mdl3=vglm(formula =

QualExam~SuffInfo+PopularInfo,data=med4.3,family=multinomial)

> summary(fit.mdl3)
```

```
Call:
vglm(formula = QualExam ~ SuffInfo + PopularInfo, family = multinomial,
    data = med4.3)

Pearson residuals:
                       Min      1Q  Median       3Q    Max
log(mu[,1]/mu[,3]) -1.0300 -0.7451 -0.6336  1.14427  1.933
log(mu[,2]/mu[,3]) -0.3834 -0.2175 -0.1656 -0.03443 14.705

Coefficients:
               Estimate Std. Error z value Pr(>|z|)
(Intercept):1 -1.524624   0.147784 -10.317  < 2e-16 ***
(Intercept):2 -1.453554   0.343240  -4.235 2.29e-05 ***
SuffInfo:1     0.114411   0.049778   2.298   0.0215 *
SuffInfo:2    -0.634753   0.155563  -4.080 4.50e-05 ***
PopularInfo:1  0.203787   0.048884   4.169 3.06e-05 ***
PopularInfo:2 -0.005186   0.148192  -0.035   0.9721
---
Signif. codes:  0 ‘***’ 0.001 ‘**’ 0.01 ‘*’ 0.05 ‘.’ 0.1 ‘ ’ 1

Number of linear predictors:  2

Names of linear predictors: log(mu[,1]/mu[,3]), log(mu[,2]/mu[,3])

Dispersion Parameter for multinomial family:   1

Residual deviance: 3074.623 on 4130 degrees of freedom

Log-likelihood: -1537.311 on 4130 degrees of freedom

Number of iterations: 7

Reference group is level  3  of the response
```

Regression with response variable "UseMon" and predictors "NotImp", "ComSubsidy", "AffCost"

```
> mdl4=read.csv("D:/V&A/Med4/Med4.3/Tab4.csv",header=T)

> attach(mdl4)

> library(VGAM)

> contrasts(mdl4$NotImp)=contr.treatment(levels(mdl4$NotImp),base=1)

> contrasts(mdl4$ComSubsidy)=contr.treatment(levels(mdl4$ComSubsidy),base=1)

> contrasts(mdl4$AffCost)=contr.treatment(levels(mdl4$AffCost),base=3)

> fit.mdl4=vglm(cbind(allsoon,later,partly)~ NotImp+
ComSubsidy+AffCost,data=mdl4,family=multinomial)

> summary(fit.mdl4)
```

```
Call:
vglm(formula = cbind(allsoon, later, partly) ~ NotImp + ComSubsidy +
    AffCost, family = multinomial, data = mdl4)

Pearson residuals:
                      Min      1Q   Median     3Q   Max
log(mu[,1]/mu[,3]) -1.545 -0.5877 -0.10027 0.9301 1.572
log(mu[,2]/mu[,3]) -1.446 -0.3369 -0.09191 0.4709 1.281

Coefficients:
                Estimate Std. Error z value Pr(>|z|)
(Intercept):1    1.86811    0.14637  12.763  < 2e-16 ***
(Intercept):2    0.91015    0.16658   5.464 4.66e-08 ***
NotImpyes:1     -0.35033    0.12946  -2.706  0.00681 **
NotImpyes:2      0.30323    0.15247   1.989  0.04673 *
ComSubsidyyes:1  0.09681    0.12895   0.751  0.45282
ComSubsidyyes:2 -0.67227    0.15075  -4.459 8.22e-06 ***
AffCosthi:1      0.69914    0.28224   2.477  0.01324 *
AffCosthi:2      0.78982    0.30123   2.622  0.00874 **
AffCostlow:1    -0.75165    0.13690  -5.490 4.01e-08 ***
AffCostlow:2    -0.91562    0.16025  -5.714 1.11e-08 ***
---
Signif. codes:  0 '***' 0.001 '**' 0.01 '*' 0.05 '.' 0.1 ' ' 1

Number of linear predictors:  2

Names of linear predictors: log(mu[,1]/mu[,3]), log(mu[,2]/mu[,3])

Dispersion Parameter for multinomial family:   1

Residual deviance: 20.3323 on 14 degrees of freedom

Log-likelihood: -67.1208 on 14 degrees of freedom

Number of iterations: 4

Reference group is level  3  of the response
```
