# Supplementary material for: Health communication, information technology and the public’s attitude toward periodic general health examinations
Source: F1000Res. 2016 Dec 30;5:2935. [Version 1] doi: 10.12688/f1000research.10508.1 (PMC5247783; doi:10.12688/f1000research.10508.1)
Supplement: Peer review history

# Peer review: Referee response for version 1

**DOI:** 10.5256/f1000research.11326.r18859

**Published:** 2017-01-13

**Authors:** Bach Xuan Tran

**Affiliations:** 1 Institute of Preventive Medicine and Public Health, Hanoi Medical University, Hanoi, Vietnam

The study findings enrich the literature on factors that influence health behaviors and health care services seeking in Vietnam. The analysis was sufficiently robust and the study has enough scientific merit for indexing.

 As for Sampling, the author may consider describing the size and development of sample frame and selection approach. If it was simple random sampling, how did the author randomly select and approach the subjects?

I have read this submission. I believe that I have an appropriate level of expertise to confirm that it is of an acceptable scientific standard.

# Response

**Published:** 2017-01-13

**Authors:** Quan-Hoang Vuong

**Affiliations:** FPT University - FSB School of Business, Vietnam

I would like to thank Professor Bach Xuan Tran for the review report and related comment. With respect to Prof Tran's suggestion on further description of the sample and the sampling practice, I find the point both valid and useful. I will seek to elaborate right when an opportunity for a data article is possible and will provide it when ready.

 Sincerely,

 Quan-Hoang Vuong

# Peer review: Referee response for version 1

**DOI:** 10.5256/f1000research.11326.r18862

**Published:** 2017-01-03

**Authors:** Cuong Viet Nguyen

**Affiliations:** 1 Institute of Public Policy and Management, National Economics University, Hanoi, Vietnam

Thank you for giving me a chance to review the paper ‘Health communication, information technology and the public’s attitude toward periodic general health examinations’. I find the paper is interesting and important for health care. In Vietnam as well as other countries, there are an increasing number of smart phones, but there is low health care utilization. The paper shows that development of reliable IT apps is a useful way to increase the health care utilization.

 The title and abstract are appropriate. The study design is well described. Overall, the paper is well written. I would like to suggest this paper for indexing.

 Best regards,

 Cuong

I have read this submission. I believe that I have an appropriate level of expertise to confirm that it is of an acceptable scientific standard.

# Response

**Published:** 2017-01-03

**Authors:** Quan-Hoang Vuong

**Affiliations:** FPT University - FSB School of Business, Vietnam

I would like to thank Professor Cuong V. Nguyen of the National Economics University (Vietnam) for your comment and especially a valid point on the utilizing of the IT devices and facilities. Given the widespread problems of cancer and diabetes in Vietnam and many other emerging market economies, where rising economic standards have not necessarily been followed by improved health and healthcare standards and the populace are subject to unequal access to quality health services, the ICT solutions appear to have been untapped. I believe the theme will further invite research efforts by both health economists and health social scientists, which would likely induce increasing interests among a broader scholarly community for the sake of bettering health for the populace.
